# Thermal Stability of Nanosilica-Modified Poly(vinyl chloride)

**DOI:** 10.3390/polym13132057

**Published:** 2021-06-23

**Authors:** Jolanta Tomaszewska, Tomasz Sterzyński, Damian Walczak

**Affiliations:** 1 Faculty of Chemical Technology and Engineering, UTP University of Science and Technology in Bydgoszcz, Seminaryjna 3, PL-85326 Bydgoszcz, Poland; damian.walczak86@gmail.com; 2 Institute of Material Technology, Faculty of Mechanical Engineering, Poznan University of Technology, PL-60965 Poznan, Poland; tomasz.sterzynski@put.poznan.pl

**Keywords:** poly(vinyl chloride), roll milling, nanosilica, thermal stability

## Abstract

The thermal stability of PVC with 1 wt % of spherical porous nanosilica, prepared by roll milling at processing time varied from 1 to 20 min, was investigated by means of visual color changes, Congo red, and thermogravimetric tests (TGA and DTG), as a function of rolling time and composition of PVC matrix. The melt flow rate (MFR) measurements were realized to identify the degradation-induced changes of processing properties. A high level of gelation of the PVC matrix for all samples was verified by DSC (differential scanning calorimetry). It was found that the addition of porous nanosilica to absorb a certain volume of HCl, produced by dehydrochlorination reaction, leads to an improvement of thermal stability, an effect observed in a form of minor color changes of the samples, lower evolution of gas hydrogen chloride, and slight changes of the MFR value. It was demonstrated that the TGA measurements are not sufficiently sensible to detect the degradation of PVC at the processing conditions, i.e., at the temperature equal to 220 °C and below this temperature.

## 1. Introduction

Poly(vinyl chloride) (PVC), due to its relatively low cost, simple processing, as well as advantageous properties such as chemical, environmental, and flammability resistance, belongs to the most applied commercial thermoplastic polymer, widely used in many industrial domains. The heat instability by processing at normal atmospheric conditions and temperatures up to 250 °C [1] presents the main restrictions by its molten-state handling. Several degradation mechanisms of PVC are proposed in the literature, but commonly, the autocatalytic dehydrochlorination reaction (zipper elimination) with subsequent formation of conjugated double-bonds sequences is assumed. The dehydrochlorination takes place at the beginning of degradation when hydrogen chloride, unfastened from the PVC chain, acts as a catalyst and accelerates the progress of decomposition, leading to formation of conjugated polyene sequences and consequently, to discoloration of PVC products. An unacceptable level of discoloration takes place even at 0.1% of dehydrochlorination. Oxidation and secondary reactions related to the presence of conjugated polyene sequences lead to chain scission, followed by crosslinking and formation of aromatic pyrolysates, an effect which is widely published [1,2,3,4,5,6,7,8]. 

Usually, the processing- and application-dependent thermal stabilizers are applied to avoid the degradation and consequently, to lower the physical, mechanical, or electrical properties of the products. Due to the broad assortment of thermal stabilizers offered on the market, the selection of an appropriate one to meet the user-specified criteria is quite a complex task, a problem vital to the production of food packaging, toys, or medical supplies.

The salts of calcium, barium, cadmium, lead, or zinc of the sulphuric, carbonic, phosphoric, stearic, palmitic, lauric, phthalic, fumaric, maleic, salicylic, and benzoic acids as well as organic compounds based on tin, organic phosphites, epoxy compounds, amino crotonates, and a-phenylindoles are currently the thermal stabilizers used by PVC processing. The stabilizers are introduced either selectively or due to an effect of stabilizing synergy, more often in the form of systems like one pack, etc. [2,3,9,10,11,12,13,14]. The stabilizing effect of the new types of compounds with a capacity to improve the thermal stability of PVC, such as zinc urate, unsaturated maleic acid–sorbitol ester, and zinc maleic acid–sorbitol ester complex, as well as mannitol stearate ester-based aluminum alkoxide was also reported [15,16,17]. This effect is often synergistic with commercially used thermal stabilizers [15,16] and other additives used in PVC blends, such as cross-linking agents and antioxidants [18].

### 1.1. PVC Composites with Mineral Additives 

Various additives including fillers are used by PVC processing, additionally to thermal stabilizers [2,19,20,21,22,23,24,25,26,27,28,29,30,31,32,33,34,35]. The impact of micro- and nanosize mineral fillers on thermal properties of PVC, including that of montmorillonite (MMT), kaolinite, hydrotalcite, and halloysite (HNT), was extensively published [2,19,20,22,23,24,25,26,27,28]. Folarin and Sadiku [2] reported the use of hydrotalcite (layered double hydroxide, LDH) for enhancing the thermal stability and lowering the smoke emission during PVC flaming. The stabilizing mechanism of LDH, such as the weakening of chlorine atoms activity and limiting the initiation of dehydrochlorination reaction, was proved by Congo red test and TGA measurements [2,23]. The thermal stabilizing effect of layered double hydroxide (MgAlLDHs) with hydroxyl groups, reacting with hydrogen chloride and restraining the progress of PVC degradation, was reported in [22].

The improvement of thermal stability of plasticized PVC was proved by using a mixture of polyhedral oligomeric silsesquioxanes (POSS) with commercial X-type zeolite (X-ZEO) and carbonate-substituted hydrotalcite (HTLC), leading to changes of decomposition profile of PVC thermal degradation [20]. 

Sodium montmorillonite (MMT) and organically modified MMT (OMMT) are frequently used as nanofillers, where their efficiency depends on the type of modifier of MMT and PVC preparation [19,24,25,26]. 

According to the TGA analysis, Gong et al. stated that the presence of organophilic MMT (OMMT) leads to lowering of the onset temperature of nanocomposites and simultaneously, to an enhancement of the rapidest decomposition temperature due to the formation of the carbonize layer on the surface of nanocomposites, thus improving the thermal stability at high temperature [24]. The lower onset of decomposition temperature (T_onset_) of the nanocomposites may be due to the influence of ammonium salt, applied to formulate the organophilic MMT via cation exchange [19,24]; a similar effect was found by Zheng et al. for plasticized PVC [25]. 

A different effect on PVC thermal stability was reported by Wan et al. [26] for MMT modified by alkyl quaternary ammonium. Based on TGA, it was found that in comparison with PVC/MMT, PVC/OMMT composites show a higher thermal stability, but at the same time, the decomposition of alkyl quaternary ammonium leads to visible discoloration of the thermally degraded PVC/OMMT composites. 

Hydrogen chloride, originated from dehydrochlorination of PVC, can be absorbed by natural and modified kaolinites, leading to increased thermal stability of PVC [27], as found by TGA measurements and color changes observation. The organophilic treatment of kaolinite may improve the thermal stability due to enhanced interactions between PVC and clay. 

A slightly improved thermal stability was found in the case of PVC modified with poly(methyl methacrylate)-grafted halloysite nanotubes (HNTs), where well-dispersed HNTs restrict the long chains mobility of PVC, so may act as a barrier, hindering the permeability of volatile degradation products [28].

### 1.2. PVC Composites with Inorganic Nanofillers

An influence of size and shape, surface characteristics, filler amount, and dispersion degree of inorganic fillers on the properties of PVC composites was published by Xie et al. [29]. Calcium carbonate (CaCO_3_), most often used as an inorganic filler, leads to an improvement of PVC thermal stability [21,29,30].

A slight increase of TGA degradation temperature by PVC/CaCO_3_ roll mill-processed nanocomposites was observed and explained by lowering of PVC long-chain mobility [29]. An influence of CaCO_3_ particle on thermal stability of PVC/CaCO_3_ composites prepared by the solution blending method was also reported by Liu et al. [21]. It was stated that the use of the 40 nm CaCO_3_ particles leads to higher thermal stability, in comparison with micrometer size particles; 1% of 40 nm CaCO_3_ particles could perform the same role as 5% of 3000-mesh CaCO_3_ particles. Chen et al. confirmed that an influence of particle size on the thermal degradation of PVC/CaCO_3_ is dominant, in comparison with CaCO_3_ concentration [30]. The total surface contact area between the CaCO_3_ nanoparticles and the PVC macromolecules is higher compared with those between microparticles and PVC, thus leading to better heat protection of the polymer matrix [30].

A similar influence of filler particle size on thermal stability of PVC was confirmed by Shimpi et al. [31], where the calcium sulfate (CaSO_4_) with 10 nm particles revealed a superior effect, in comparison with bigger particles, such as 24 and 17 nm. Consequently it was suggested that the use of minor-size filler particles, resulting in a smaller distance to PVC macromolecules, may better restrain the evaporation flow of volatiles from the decomposed material.

### 1.3. Silica Nanofillers as Modifiers of PVC Composites

Although the PVC thermal stability with nanoscale fillers has been widely studied, the use of nanoscale silica has rarely been published. 

Chen et al. has reported that the TGA-determined thermal stability of rigid PVC nanocomposites with untreated and surface-modified nanosilica is lower than that of neat PVC. However, modification of SiO_2_ particles by silane coupling agent and additionally by ultrasonic oscillations and high-energy vibromilling leads to an improvement of thermal stability of PVC composites, particularly at high temperatures (400–500 °C), in comparison to untreated nano-SiO_2_. A final conclusion was proposed that untreated SiO_2_ particles may initiate the dehydrochlorination of PVC, while SiO_2_ particles coated by an organic compound do not initiate this process [32].

It was also found that the temperature of the initial step of the thermal degradation process of PVC composites depends on the size of silica particles. The T_onset_ value equal to 225 °C was noted for SiO_2_ particle with 25 nm size, and a value of 228 °C when Si particles with 1.5 μm size were used [33].

The influence of SiO_2_ particles concentration with 20 nm size on properties of thermally aged PVC was published in [34]. Thermal treatment by 110 °C and by 140 °C allowed to assign the 7.5 wt % SiO_2_ concentration as a composition with lower mass-loss rate, in comparison with neat PVC. It was also stated that the power cables with isolation made of PVC/SiO_2_ nanocomposite have a longer lifetime, compared to cables isolated by neat PVC.

We have studied the influence of lignin (L) combined with 3.5 µm silica particles (S) on the PVC thermal properties [35]. Although the degradation behavior of PVC/S/L composites, indicating two major TGA stages of decomposition, is similar to that of pristine PVC, the values of temperature corresponding to mass loss of 1%, 5%, and 50% are higher, compared with those for unfilled PVC. With a silica content (as a separate component) of 7.5 wt % in the PVC matrix, a decrease of TGA temperature of 5 to 9 °C, measured by 1% mass loss, was observed, an effect evident in the initial stage of PVC decomposition. An influence of silica on the decomposition temperature at further stages of degradation was not found. The results of Congo red thermal stability test confirmed that the addition of silica causes a significant drop in the thermal stability of the PVC matrix, especially by 5 and 7.5 wt % concentration.

The influence of additives on the thermal stability of PVC has been investigated by various measurements techniques, presented schematically below. 

The Congo red test [16,17,23,36] and thermal gravimetric analysis [14,17,24,25,26,27,28,29,30] were used to determine the thermal stability of PVC in static conditions. The use of Brabender or Haake torque rheometers allows to measure the thermal stability in dynamic conditions [16,17,25,37,38].

The detection of other effects, such as color changes due to hydrogen chloride evaporation [17,23,25,26,27,36,37,39] and/or fluctuations in rheological properties due to degradation of PVC molecular structure [37,38], may also be used as indirect methods to study the degradation progress.

The thermal stability and the problem of potential thermal degradation of PVC by processing is well known, presenting certain limits for the industrial production of PVC products. The knowledge of PVC thermal stability is substantial regarding the selection of appropriate conditions, allowing to avoid the risk of thermal decomposition during melt processing. This effect is particularly essential if the production of PVC products by means of conventional processing techniques, like extrusion and/or injection molding, is applied. The influence of processing temperature and shearing in the melt is widely described in the literature [40,41,42,43,44,45,46].

The processing time-induced changes of thermal stability of the PVC/SiO_2_ nanocomposites are the main topic of our research. The detection of the degradation process and its outcomes observed in the form of significant color changes as a function of roll-milling ageing time, as well as the processing-related detection of gelation degree by thermal investigations, should support also the future-oriented estimation of the influence of nanosilica on PVC thermal stability. The aim of our research was to detect the influence of a low quantity of nanosilica on the thermal degradation of the poly(vinyl chloride) and to quantify these effects by means of various investigation techniques. 

## 2. Materials and Methods

### 2.1. Materials 

Two types of PVC compounds as polymer matrix were used in our research. The first one, marked as PVC_R_ (reference compound), was a dry blend composed of 100 phr suspension PVC S-61 Neralit (Spolana Anwil Group, Neratovice, Czech Republic), 4 phr organotin stabilizer Patstab 2310 (Patcham, Goor, The Netherlands), and 1 phr Naftolube FTP paraffin wax (Chemson, Arnoldstein, Austria). The stabilizer and the paraffin wax were the only added components in order to minimize the influence of additives on processing properties.

The second PVC compound, denoted as PVC_C_ (commercial compound), was a commercially available nonplastified PVC S-58 dry blend (a compound fully formulated and donated by Anwil S.A., Włocławek, Poland) according to [47] with additives such as Ca/Zn thermal stabilizer, acrylic impact modifier, and acrylic processing aid, as well as external and internal lubricant.

The nanosilica, in the form of spherical, porous nanopowder (delivered by Sigma Aldrich, St. Louis, MO, USA) with an average dimension between 5 and 15 nm and purity 99.5%, was introduced in a concentration of 1 wt %, as nanofiller into the PVC matrix. 

The nanosilica porosity was determined by using the measurement technique proposed by Klapiszewski et al. [35]. The surface area of nanosilica was determined by multipoint BET (Brunauer–Emmett–Teller) technique, using data for adsorption under relative pressure (p/po). The total pore volume and mean pore size were evaluated according to BJH (Barrett–Joyner–Halenda) algorithm. The surface area measurements were realized by means of an ASAP 2020 instrument (Micromeritics Instrument Co., Norcross, GA, USA), with adsorptive characterization, by three measurements performed for each sample. The surface area was determined with a precision of ±0.1 m^2^/g, the pore volume ±0.01 cm^3^/g, and the pore size ±0.01 nm. 

It was found that the surface area of the nanosilica was 393.6 m^2^/g, the total pore volume was 0.51 cm^3^/g, and the average pore size 5.22 nm. 

### 2.2. Processing

The PVC compounds were produced by means of a roll mill (Buzuluk Inc., Komarov, Czech Republic) with roll length 38 cm, diameter 20 cm, operating at 170 °C, with the rolls’ rotation rate of 0.35 and 0.40 s^−1^, the slit between the rolls was 1.5 mm and the friction 1:1.2. The samples of neat PVC compounds and nanosilica-modified compounds were investigated after processing times of 1, 5, 10, 15, and 20 min, with the exception of PVC_C_ compound processed during 20 min due to its significant thermal degradation. The start of the sampling time began with the visual appearance of a homogenous gelation of the rolled sheet. 

### 2.3. Measurements

#### 2.3.1. Gelation Level by DSC

The gelation degree was determined by differential scanning calorimetry (DSC) based on the registration of thermal transition, corresponding to melting of primary and secondary crystallites. The Phoenix DSC 204 F1 apparatus (Netzsch, Selb, Germany) operating at temperature range between +50 and +240 °C, with a heating rate of 10 °C/min, was used. The nitrogen served as an inert atmosphere, and the average weight of the sample was 15 to 20 mg. The gelation degree (*G*) was estimated according to the formula proposed by Potente [41], applied by Zajchowski [42], based on the melting enthalpy (in J/g) of the primary (∆*HA*) and secondary (∆*HB*) crystallites:

(1) $$G=\frac{\Delta HA}{\Delta HA+\Delta HB}\times 100\%$$

where Δ*HA*—the melting enthalpy of the secondary crystallites (J/g) and Δ*HB*—the melting enthalpy of the primary crystallites (J/g).

#### 2.3.2. Thermal Stability

The thermal stability of PVC and PVC/SiO_2_ nanocomposites was investigated by visual observation, Congo red test, thermogravimetric analysis (TGA), and melt flow rate (MFR) measurements.

To detect the PVC degradation-induced changes of coloration after defined rolling times of 1, 5, 10, 15, and 20 min, the samples were taken out, and a set of photos was completed. 

The thermal stability test using Congo red method was carried out according to ISO 182-1:1990 standard. The samples were heated in the oil bath at 200 °C, and the time of thermal stability related to an evolution of hydrogen chloride gas, leading to changes in color of the indicator to about pH3, was measured.

The TGA measurements were performed using an analyzer TG 209 F3 (Netzsch, Selb, Germany) operating at a scanning rate of 15 °C/min, under nitrogen protective atmosphere, in the temperature range from 30 to 550 °C. The composite samples with a weight of about 10 to 15 mg were taken from roll sheet at the defined processing time and placed in the ceramic crucible. For every composition, three TGA measurement procedures were repeated. The temperature of 1% weight loss (T_1_) and the temperature of the maximal degradation rate (as a maximum value at the DTG graph) were analyzed. 

#### 2.3.3. MFR Measurements

The processing properties of both PVC dry blends and compounds with nanosilica were determined by means of MFR technique, operating at the following experimental conditions: the standard die of 8 ± 0.025 mm in length and 2.095 ± 0.005 mm in diameter, piston load 400 N, and time of polymeric samples heating equal to 10 min. Due to the different composition of the PVC samples, the temperature of the cylinder was 175 °C for PVC_C_ and 190 °C for PVC_R_, respectively. 

#### 2.3.4. The Method of Color Measurement 

The color of the samples was measured by CIE L*a*b* procedure, by means of colorimeter type Chroma Meter Konica Minolta CR-410 (the light source D65, observer 2°). The parameter L* denotes the lightness of the color, with the values between 0 and 100 (%). The coordinate a* with a positive value signifies the red color existing in the measured object, and with negative value it signifies the green color of the spectrum. The positive value of parameter b* is characteristic for the yellow color at the spectrum, and the negative value means the presence of blue coloring. The measurements were realized at least three times for each sample, and the average values were analyzed. The relative changes, Δ$L_{SiO{}_{2}}^{*}$ (%), were characterized as a difference of lightness between the samples of PVC with SiO_2_ ($L_{PVC/SiO{}_{2}}^{*}$) and those without SiO_2_ ($L_{PVC}^{*}$), referenced to lightness of a sample without SiO_2_, respectively, for reference and commercial PVC. The relative changes of the L* were estimated by following formula:

(2) $$\Delta L_{SiO{}_{2}}^{*}=[(\;L_{PVC/SiO{}_{2}}^{*}-L_{PVC}^{*})/L_{PVC\;}^{*}]\times 100\%$$

The relative changes Δ$L_{proc}^{*}$ (%) were evaluated as a difference between lightness L* of PVC or PVC/SiO_2_ processed in various rolling times ($L_{time}^{*}$) and L* of PVC or PVC/SiO_2_ taken after 1 min ($L_{\;1\min}^{*}$) referenced to lightness of PVC or PVC/SiO_2_ taken after 1 min, according to the following formula:

(3) $$\Delta L_{proc}^{*}=[\left(L_{time}^{*}-L_{\;1\min}^{*}\right)/L_{1\min}^{*}]\times 100\%$$

## 3. Results

### 3.1. The DSC-Determined Gelation Level

**Figure 1:**
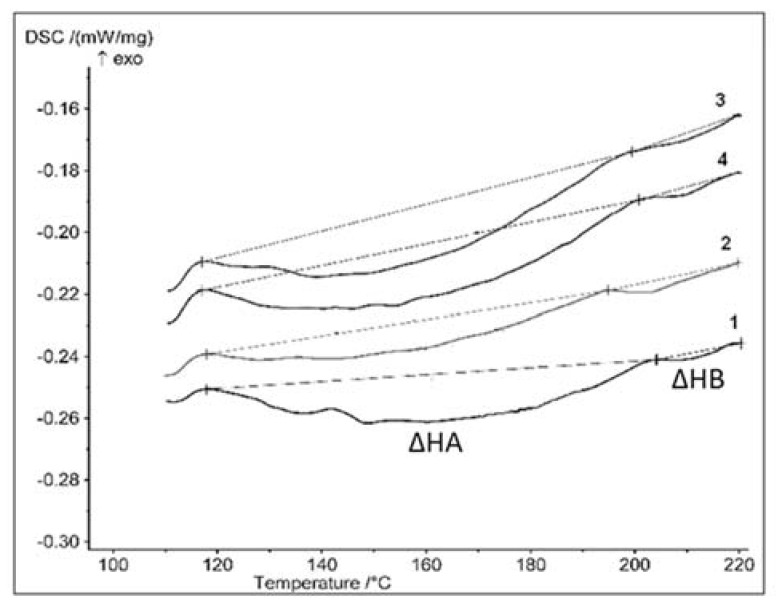
DSC thermograms for PVC_R_ and PVC_R_/SiO_2_ composites processed in rolling time of 1 min and 20 min: (1) PVC_R_ 1 min; (2) PVC_R_ 20 min; (3) PVC_R_/SiO_2_ 1 min; (4) PVC_R_/SiO_2_ 20 min.

According to the literature, the gelation process of poly(vinyl chloride) consists of transformation of the grains’ morphology, depending on temperature and shearing [41,42,43,44,45,46]. The DSC investigations were realized for PVC compounds without and with nanosilica, for samples after 1 and 20 min of processing by roll milling. The corresponding DSC thermograms for PVC_R_ and PVC_R_/SiO_2_ are presented on Figure 1. The endotherms related to melting of primary crystallites (ΔHB), followed by creation of secondary crystallites structure (ΔHA), may be seen on the thermograms. As these endothermic peaks represent a relatively high melting enthalpy, followed by an essentially smaller peak of the melting of primary crystallites that did not undergo transformation during processing, a high degree of gelation may be concluded. The values of gelation degree, evaluated according to Equation (1), are summarized in Table 1.

In the case of both nonmodified PVC compounds, the gelation degree is relatively high; for the samples after 1 min of rolling time, the corresponding values for PVC_C_ and PVC_R_ are 88% and 91%, respectively. 

Processing of the PVC_R_ sample for another 5 min resulted in a 5% rise of gelation degree, followed by an increase of G value by about 1% to 2%, by 20 min of rolling time. The addition of nanosilica to the PVC_R_ compound permits to achieve a very high value of gelation level of about (98%), by rolling of 1 min, starting at the forming ofa homogenous material, which remains constant for a longer rolling time. 

An increase of the gelation degree by 6% for commercial PVC_C_ compound was observed for 5 min processing time, followed by a rise of G value to 98–98%. Quite a similar run of rolling time dependence of gelation degree, however for slightly lower G values, was found for the nanosilica-modified PVC_C_. The G value of PVC_C_ processed for 20 min could not be determined due to visible degradation of the polymer.

### 3.2. Melt Flow Ratio of PVC and PVC with Nanosilica

**Figure 2:**
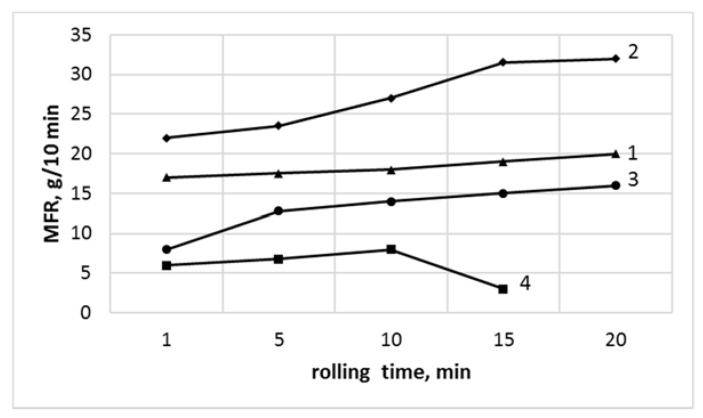
The MFR of PVC and nanocomposites with SiO_2_ as a function of rolling time: (1) PVC_R_/SiO_2_; (2) PVC_R_; (3) PVC_C_/SiO_2_, and (4) PVC_C_.

The influence of the rolling time on the MFR values is presented in Figure 2.

The samples for the MFR were taken after the same rolling time as for the red Congo test. Two various MFR measuring temperatures were applied, i.e., 175 °C for the PVC_C_ and 190 °C for the PVC_R_, since the commercial PVC includes the melt flow modifier, not present in PVC_R_. Consequently, the MFR values of PVC_R_ samples are always significantly higher, compared with those of PVC_C_ compounds (Figure 2, curve 1,2). 

The judgement of nanosilica influence on PVC composite properties was demonstrated. As it follows from Figure 2,the influence of nanosilica on the processing properties of each PVC compound varied, i.e., a slight increase of the MFR value of PVC_C_ compound, thus an improvement of the processing ability (curve 3), and a decrease of the MFR in the case of PVC_R_ (curve 1). These observations are valid for all rolling times applied. 

For a longer milling time, an increase of MFR value is related to degradation effect; thus, to lowering of the macromolecular mass of the polymers (Figure 2, curve 1, 2, and 3). A significant decrease of MFR value of nonmodified PVC_C_, observed for a milling time of above 10 min, allows to suggest the degradation, which may be accompanied by a cross-linking of the polymer, as a dominating effect during PVC processing (Figure 2, curve 4). Accordingly, it may be stated that in the case of the PVC_C_, the incorporation of SiO_2_ leads to significantly higher thermal stability and simultaneously to better processing properties. 

A considerable increase in MFR value of PVC_R_ without nanosilica, compared to PVC_R_/SiO_2_ compound, along with the milling time may be explained by the lowering of macromolecular mass, due to the processing-induced degradation. An almost constant value of MFR, independently of the milling time, was observed for PVC_R_+SiO_2_ compound. As mentioned before, a positive influence of nanosilica on thermal stability of PVC_R_ in the form of its lower dynamic of degradation process was established. However, from the other side, the addition of SiO_2_ led to lowering of MFR value of PVC_R_/SiO_2_ compounds, thus to partially less advantageous processing properties. 

### 3.3. The Relationship between MFR and DSC

The progress of gelation may indirectly be analyzed by changes in MFR values, as well as by DSC testing of thermal effect-related changes of primary and secondary structure elements. Due to the creation of secondary structures and simultaneous disappearance of the grain morphology, an increase in G level is usually accompanied by a decrease in the MFR value [42,45]. 

**Figure 3:**
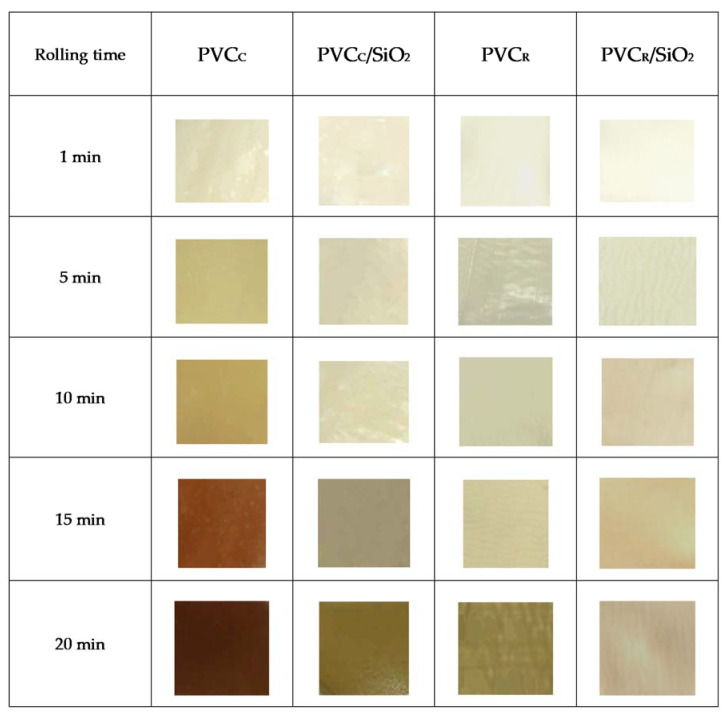
Color changes of PVC_C_ and PVC_R_ samples and PVC composites with SiO_2_ as a function of the time of rolling progress.

**Figure 4:**
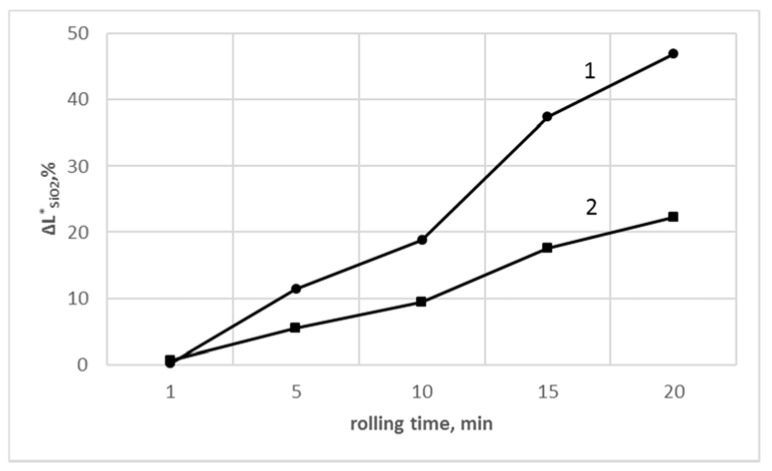
The relative change, Δ$L_{SiO{}_{2}}^{*}$ (%), values of SiO_2_-modified PVC vs. unmodified PVC, as a function of rolling time: (1) PVC_C_/SiO_2_ vs. PVC_C_; (2) PVC_R_/SiO_2_ vs. PVC_R_.

It is known [41,42,43,44,45,46] that gelation is essentially dependent on processing temperature and shearing, existing in molten flow. In our case, it was confirmed that the PVC handling time, with constant processing conditions of temperature and mechanical charges, plays a significant role (Figure 1, Figure 3, and Figure 4). The DSC-evaluated gelation level is high for all samples (above 84%) (Table 1), signifying the transformation of primary structure of PVC grains during processing [41,42,43,44,45,46]. Consequently, a lowering of MFR values was not observed in this case (Figure 2), although it was an effect described in our primary investigations [42,45]. 

As a high gelation degree was observed after a relatively short time of milling processing, i.e., after about 1 min, the following changes of the MFR value may be ascribed to time-dependent degradation of the macromolecular structure of PVC. Therefore, the MFR results may prove the influence of nanosilica addition on the modification of the thermal stability of investigated PVC systems. 

### 3.4. Visual Observation of PVC Degradation

In order to determine the degradation-induced changes of coloration, the samples were taken from the rolled PVC sheet at a defined time and presented as a set of photos (Figure 3). 

The temperature higher than PVC softening temperature, i.e., above 150 °C, may induce several chemical reactions, such as dehydrochlorination, oxidation, and degradation. Particularly, due to the dehydrochlorination, the appearing double bonds may lead to color changes of the PVC samples; in this case, the yellowness was followed by dark red and finally by black color, an effect strongly dependent on heating time [1,2,3,4,5,6,7,8,17,18,23,25,26,27,36,37,39].

The PVC_R_ samples processed in our laboratory showed significantly higher thermal stability compared with the PVC_C_ sample, which may be seen in the degradation time-dependent changes of color (Figure 3). Once the roll milling process of PVC_R_ attained 15 min, its color changed to yellow and a dark brown coloration may be observed at PVC_C_. This result indicates a highly advanced polymer degradation, i.e., a lower thermal stability of this compound, due to various composition of PVC_R_ and PVC_C_ compounds, particularly owed to the different type and loading of the thermal stabilizers. Analogous results of color changes were observed by Brostow et al. [18] for the plasticized PVC as a function of radiation time. 

Similar observations of degradation-induced color changes were published in papers where the stabilizing effectiveness of various additives, including maleic acid–sorbitol ester and zinc maleic acid–sorbitol ester complex [17], zinc orotate [36], layered and intercalated hydrotalcite [23], montmorillonite [25], kaolinite [27], and other nanoclays [39], was evaluated using discoloration test. 

Both PVC_C_ samples, with and without SiO_2_, taken after 1 min of rolling (beginning at homogenous gelation) demonstrated a similar slight change of coloration. However, for a longer processing time, a lighter coloration of samples with nanosilica, compared with samples without nanofillers, may be seen, an effect indicating a role of this powder filler by enhancement of PVC thermal stability. After processing time of 10 or 15 min, a dark brown color of nonmodified PVC_C_ was noticeable, on the contrary, a grey color of the PVC_C_/SiO_2_ samples was visible, indicating an improved thermal stability of the silicium-modified nanocomposites. Consequently, it may be concluded that the nanocomposites containing SiO_2_ may support a longer processing time, in comparison with nonmodified PVC_C_. 

In the case of the reference PVC_R_ samples, only a slight discoloration may be observed, nearly independent of the processing time. Although the color changes for the PVC_R_ and PVC_R_/SiO_2_ were not significant, the nano-additives-modified poly(vinyl chloride) was more thermally stable, i.e., its visual degradation was observed after longer processing time. Further, a slightly more intense difference of coloration between SiO_2_-modified and nonmodified PVC was visible for the roll milling time longer than 10 min (Figure 3). 

### 3.5. Determination of Color Parameters 

The color of both series, i.e., the values of L*, a*, and b*, of PVC_R_ and PVC_C_, with and without nanosilica, was rolling time-dependent, where due to the addition of SiO_2,_ these variations are different (Table 2 and Table 3). 

After 1 min of rolling, an equal value of parameter L* for the PVC_C_ and PVC_C_/SiO_2_ was observed. For an extended rolling time, a lowering of the lightness L* was noted, where the L* values are always superior for the corresponding samples containing the nanosilica additive (Table 2).

The color quality is changing as well, i.e., the b* value was increasing (by PVC_C_ processed up to 10 min, and by PVC_C_/SiO_2_ to 20 min), indicating a rising of the yellow component. Depending on the PVC_C_ rolling time, the participation of the green component was decreasing; after 5 min, a positive value of a* parameter, i.e., the red part of the color, was noted. In the case of PVC_C_/SiO_2_ sample, this alternation was observed first after 15 min of processing. 

The addition of SiO_2_ in the case of PVC_R_ resulted in a higher L* value, independent of the processing time, meaning that the PVC_R_/SiO_2_ samples were always luminously, compared with nonmodified PVC samples. Similarly, as for the PVC_C_, the value of the b* parameters for the PVC_R_ samples are positive, increasing as a function of the rolling time. The positive value of a* indicates growing of the red color component, observed after 20 min of processing for the PVC_R_ sample and after 10 min of processing of the PVC_R_/SiO_2_ material (Table 3). 

The relative changes of Δ$L_{SiO{}_{2}}^{*}$ (according to Equation (2)) of PVC samples without and containing SiO_2_ for PVC_R_ and PVC_C_ compounds as a function of the milling time are presented in Figure 4. Longer rolling times led to higher the Δ$L_{SiO{}_{2}}^{*}$ values. As it may be seen, the nanosilica had a protective influence on the color changes even after 20 min of processing in the case of the modified PVC samples. 

The difference of lightness between the PVC and PVC + SiO_2_ materials is significantly higher for the PVC_C_ samples, indicating a superior efficiency of nanosilica on the improvement of thermal stability of these samples. This effect was also found by the determination of the relative changes of thermal stability of SiO_2_ modified PVC vs. unmodified PVC measured by means of red Congo test.

**Figure 5:**
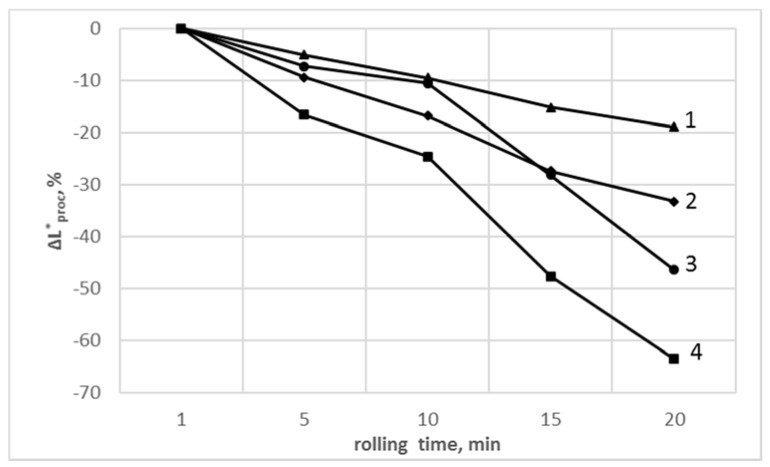
The relative change of ΔL*_proc_ values of PVC and PVC/SiO_2_ processed in various rolling times vs. PVC and PVC/SiO_2_ taken after 1 min as a function of processing time: (1) PVC_R_/SiO_2;_ (2) PVC_R_; (3) PVC_C_/SiO_2_; (4) PVC_C_.

As it follows from Figure 5, the relative change ΔL*_proc_ values (according to Equation (3)) of all samples referenced to L* values corresponding to those processed during 1 min was decreasing independent of sample composition. Lowering of ΔL*_proc_ values was more pronounced for PVC_C_ material, both for nonmodified and SiO_2_-modified compound.

Based on the above-mentioned results, it may be stated that the addition of nanosilica plays a more significant role by improving the thermal stability of PVC_C_ samples. 

### 3.6. The Thermal Stability Determined by Congo Red

**Figure 6:**
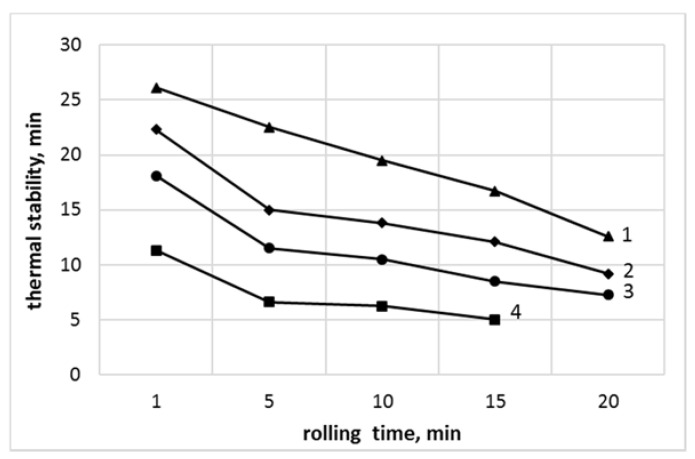
Congo red thermal stability of PVC and nanocomposites with SiO_2_ as a function of rolling time: (1) PVC_R_/SiO_2_; (2) PVC_R_; (3) PVC_C_/SiO_2_, and (4) PVC_C_.

The Congo red thermal stability as a function of the rolling time is presented in Figure 6.

An adequate agreement between the processing time-dependent discoloration and the results of the Congo red test was observed. From Figure 6, it follows that independent of the rolling time, the thermal stability was always higher for the PVC_R_ compared with the PVC_C_ samples, an effect resultant from various thermal stabilizers used in both compounds. An improved thermal stability was also observed as a consequence of nanosilica application. For example, in the case of PVC_C_/SiO_2_, a 20 min processing time resulted in the coloration similar to that observed for nonmodified PVC_C_ samples examined after 5 min of milling time. A similar tendency was noted for PVC_R_/SiO_2_ where the level of degradation, observed after 20 min of processing, seems to be similar to that of PVC_R_ after 15 min of rolling time. 

**Figure 7:**
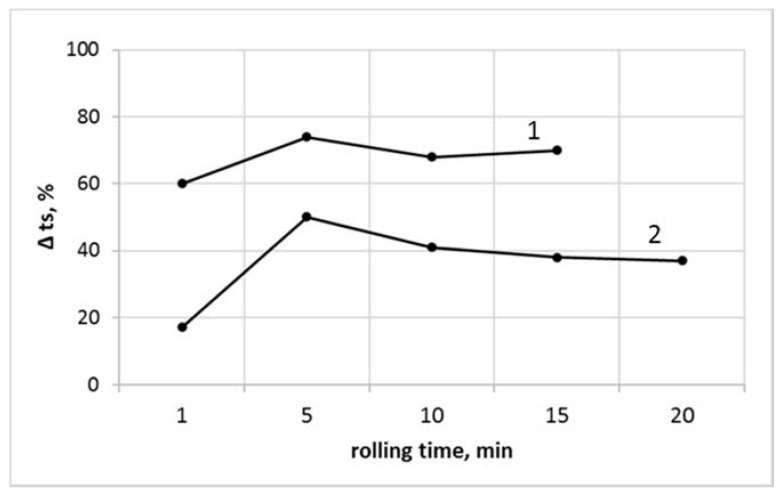
The relative changes of thermal stability of SiO_2_-modified PVC vs. unmodified PVC, as a function of rolling time: (1) PVC_C_/SiO_2_ vs. PVC_C_; (2) PVC_R_/SiO_2_ vs. PVC_R_.

The relative values of thermal stability of samples containing SiO_2_, referenced to thermal stability of nonmodified PVC_R_ and PVC_C_ compounds (Δts), as a function of the milling time are presented in Figure 7. 

In all cases, an improved thermal stability of nanosilica-modified compounds, independent of the time of shearing during melt milling, was seen. A high improvement of Δts value of about 60% to 74% was noted for the commercial blend with nanosilica (PVC_C_/SiO_2_), in comparison with PVC_C_. An efficiency of nanosilica-induced improvement of thermal stability was significantly lower in the case of the PVC_R_ compound initially modified only with lubricant and thermal stability additives, where the relative increase of the stability time was equal to 17% to 50%. It was also found that the efficiency of the nanosilica addition was particularly important during the first 5 min of processing time. For longer milling time, a reduced improvement of thermal stability was observed, an effect which may be related to lower intensity of HCl releasing from degraded PVC macromolecules and/or to limited gas absorption opportunity of the porous nanosilica, arising probably after several minutes of high absorption activity. 

### 3.7. Thermogavimetric TGA Measurements

**Figure 8:**
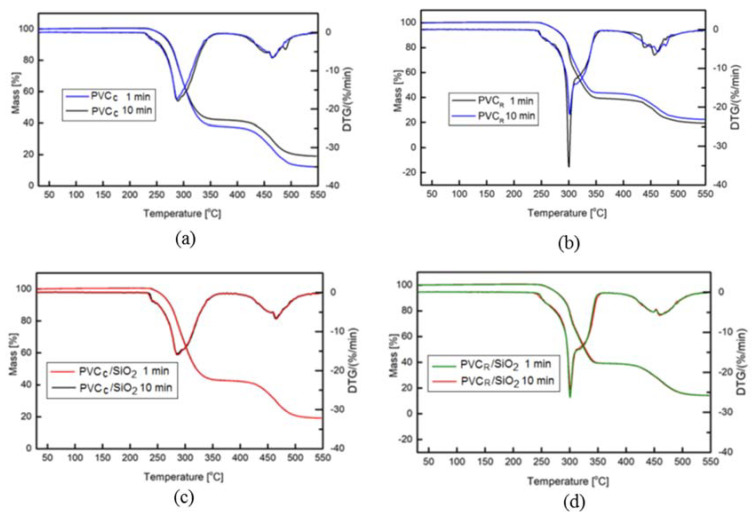
TGA/DTG thermograms of (**a**) PVC_C_; (**b**) PVC_R_; (**c**) PVC_C_/SiO_2_, and (**d**) PVC_R_/SiO_2_ processed in 1 and 10 min of rolling time.

Habitually, the processing time of PVC by means of industrial machines and equipment (so-called residence time) is usually no longer than several minutes, thus the TGA detection of degradation was examined only during 1 and 10 min. The TGA and DTG curves recorded in the temperature range between 50 and 550 °C are presented in Figure 8a–d. The degradation trend of PVC nanocomposites is similar to that of unfilled PVC, and consists of two major steps. The first degradation stage observed in the temperature range of 230–350 °C is assigned to the progressive dehydrochlorination of PVC and to the formation of conjugated polyene structure. The second stage, occurring in higher temperature range, corresponds to thermal cracking of the carbonaceous conjugated polyene sequences and the formation of residual chars [48].

As the aim of our research was to describe the influence of nanosilica on the matrix degradation in the PVC processing range, we have discussed the effects observed at the temperature array limited to the beginning of the mass—TGA changes, corresponding to 1 wt % of the mass loss (Δm).

As it follows from Figure 8a–d, at temperatures up to about 220 °C, any significant changes of the TGA run were observed. Both the run of mass changes as a function of temperature (TGA) and the run of the mass changes rate (DTG) demonstrate constant values, signifying that at this temperature, the degradation effects are not detectable by the TGA measurement. This outcome may be explained by primary processing realized in various times, contrary to the results published in [24,25,26,27,28,29,30] where the modification of PVC matrix was done only by addition of montmorillonite [24,25,26], kaolinite [27], halloysite [28], and calcium carbonate [29,30] without long-time shearing of molten PVC generating certain thermal degradation. 

First, at a temperature above 240 °C to 250 °C, a decrease of mass was observed in a form of lowering of Δm values and as increased values of Δm/ΔT. The temperature by defined Δm, is given as a characteristic of the thermogravimetric measurements. Our interest was to study the degradation at the PVC processing range; thus, in Table 4, the corresponding values of temperature T_1_ by 1% of mass loss are shown. It may be seen that for all studied composites, higher values of T_1_ were always observed for the PVC_R_ compound, in comparison with commercial PVC, independent of the processing time (1 and 10 min) and the addition of nanosilica, an effect due to various stabilization composition applied in both PVC compounds. 

An enhancement of thermal stability of PVC nanocomposites with silica may be observed, as noted by Congo red test. In the case of the reference samples, an increase of the T_1_ values of about 2 to 3 °C due to the SiO_2_ addition was noted, a somehow lower increase of T_1_, of about 1 to 2 °C, was observed for commercial samples. With higher TGA investigations temperatures, the influence of SiO_2_ on the thermal stability of the PVC matrix was not confirmed, as probably the dehydrochlorination effect was predominant, and the HCl was be absorbed by the nanosilica, due to the relatively low content of SiO_2_ of about 1 wt %. 

The maximum at the DTG curves signifies the temperature T_DTG_ where the degradation of the polymer is the most intensive, although this thermal effect is well above the industrial processing domains of PVC compounds. Nevertheless, we have found (Table 4) that also in this case, the thermal degradation of the commercial PVC compound appeared at the temperature lower than about 10 to 15 °C, in comparison with the reference samples. In both cases, the influence of nanosilica was not evident, due to the effect mentioned above, i.e., a very high HCl flow, which may not be absorbed by the SiO_2_, even regarding its porous character.

## 4. Summary

The DSC-determined gelation degree for all investigated PVC samples was found to be relatively high, even after a short time of handling, thus indicating the ability to process the PVC compounds in optimal conditions. Owing to a high gelation degree, signifying the transformation of morphology structure elements, a usually existing decrease of the MFR value was not observed; thus, a prevailing role of time-dependent macromolecular chains degradation was proposed to explain this effect. Accordingly, a certain increase of the MFR value may be ascribed to lowering of the PVC macromolecular mass, although in certain cases, the partial cross-linking of nonmodified samples led to lowering of the MFR value.

The incorporation of nanosilica with porous structure allowed to improve the thermal resistance of PVC compounds, due to the absorption effect of the released HCl gas, in agreement with other authors [49,50]. Particularly, a higher efficiency of nanosilica was noted for the PVC_C_ samples with significantly lower thermal stability (Figure 4 and Figure 7) compared with PVC_R_. Another attainment of our studies is that the most effective stabilization activity of the SiO_2_ was observed at the beginning of processing. 

It was ascertained that in addition to the composition, the degradation process is strongly related to processing time. The limited ability of the porous nanosilica to absorb the HCl gas, as a direct product of the PVC dehydrochlorination, may be the reason for this observation. As a corroboration of this effect, the time-dependent changes of the color of the samples may be considered, where in the case of silica—modified poly(vinyl chloride), the progress of discoloration effect was always slower, compared with observation of corresponding nonmodified PVC probes. These explanations were confirmed by lightness measurements. The tests of color changes influenced by thermal degradation are often used in the case of assessment of stabilization efficiency of various PVC compositions [17,18,23,25,27,36,39].

Although the thermogravimetric measurements usually deliver data showing the mass lowering by thermal degradation of polymers, relatively low values of mass loss at the processing temperature domain due to the dehydrochlorination were noted in our case. Nevertheless, a slight improvement of TGA-determined stability of the SiO_2_-modified PVC samples was found.

## 5. Conclusions

1. Various thermal effects related to PVC degradation may be specified by means of Congo red test, color changes, and melt flow ratio measurements.
2. The time-dependent discoloration of PVC samples signifies the progress of degradation related to dehydrochlorination. The use of SiO_2_ nanoparticles affects the time-related degradation and thus the time to observe the evolving of HCl.
3. A significant role of processing conditions, particularly the rolling time, on the macromolecular degradation was stated by MFR measurements.
4. The porosity of nanosilica plays an important role by immobilization of dehydrochlorination products, thus improving the thermal stability of PVC/SiO_2_ systems, an effect observed mainly at the beginning of thermal degradation due to limited volume of the pores.
5. It has to be stressed that the stabilization role of SiO_2_ nanoparticles is related not only to PVC composition but also to type and conditions of its processing.

## Figures and Tables

**Table 1:** Degree of gelation (%) of PVC_R_ and PVC_C_ and PVC nanocomposites with SiO_2_.

Rolling Time, min	PVC_R_	PVC_R_/SiO_2_	PVC_C_	PVC_C_/SiO_2_
1	91	98	88	84
5	96	98	94	93
10	97	97	98	95
15	98	97	99	97
20	98	97	-	98

**Table 2:** Values of lightness L* and a*, b* coordinates for commercial PVC without SiO_2_ (PVC_C_) and with SiO_2_ (PVC_C_/SiO_2_).

Sample	PVC_C_			PVC_C_/SiO_2_		
Rolling time	L*	a*	b*	L*	a*	b*
1 min	87.33	−2.85	9.27	87.49	−2.81	5.30
5 min	72.8	0.71	18.955	81.10	−2.10	9.83
10 min	65.85	5.03	26.43	78.26	−1.13	6.21
15 min	45.72	19.06	13.51	62.83	3.34	8.35
20 min	31.87	6.22	0.5	46.84	5.99	12.55

**Table 3:** Values of lightness L* and a*, b* coordinates for reference PVC without SiO_2_ (PVC_R_) and with SiO_2_ (PVC_R_/SiO_2_).

Sample	PVC_R_			PVC_R_/SiO_2_		
Rolling time	L*	a*	b*	L*	a*	b*
1 min	85.51	−1.19	0.09	86.09	−2.00	0.16
5 min	77.48	−2.34	5.54	81.72	−2.775	4.86
10 min	71.16	−2.58	8.49	77.89	0.69	9.03
15 min	62.09	−0.91	12.6	72.99	1.08	16.04
20 min	57.08	4.88	20.61	69.77	4.55	8.51

**Table 4:** The temperature of 1% weight loss (T_1_) and the temperature of the maximal degradation rate (T_DTG_) for the PVC_R_, PVC_R_/SiO_2_, PVC_C_, and PVC_C_/SiO_2_ processed in 1 and 10 min of rolling time.

Sample	T_1_, °C	T_DTG_, °C
Reference PVC		
PVC_R_, 1 min	255.9	300.0
PVC_R_, 10 min	254.3	301.9
PVC_R_/SiO_2_, 1 min	258.2	300.7
PVC_R_/SiO_2_ 10 min	257.5	301.6
Commercial PVC		
PVC_C_, 1 min	245.6	289.4
PVC_C_, 10 min	243.9	288.1
PVC_C_/SiO_2_, 1 min	246.6	285.1
PVC_C_/SiO_2_, 10 min	245.6	285.8

## Data Availability

There are no specific reports with data availability.
